# Clinical symptoms of COVID-19 pneumonia in children: A protocol for systematic review and meta-analysis

**DOI:** 10.1097/MD.0000000000024108

**Published:** 2021-01-08

**Authors:** Zhengwu Tang, Muzhe Li, Wei Chen, Xun Ran, Huiyun Li, Zhiwei Chen

**Affiliations:** Department of Orthopaedics, The First Affiliated Hospital of University of South China, Hengyang, China.

**Keywords:** COVID-19, meta-analysis, nonsevere, severe

## Abstract

**Background:**

This meta-analysis aimed to compare the clinical symptoms of COVID-19 pneumonia in children.

**Methods and analysis:**

Electronic databases including PubMed, EMBASE, Web of Science, China National Knowledge Infrastructure (CNKI) database, Wanfang Database, and Chinese Biomedical Literature Database (CBM) were searched from its inception to June 21, 2020. We only included studies that reported clinical symptoms of COVID pneumonia in children. Quality of the included studies was assessed by 2 authors. Pooled results were summarized by STATA 12.0 software.

The heterogeneity was measured by *I*^2^ tests (*I*^2^ < 50 indicates little heterogeneity, *I*^2^≥50 indicates high heterogeneity). Publication bias was performed by funnel plot and statistically assessed by Begg test (*P* > .05 as no publication bias).

**Results:**

Results will be shown as figures or tables.

**Conclusion:**

Our study aims to systematically present the clinical symptoms of COVID-19 pneumonia patients in children, so as to further provide guidance for clinical management.

## 1 Introduction

Coronavirus disease 2019 (COVID-19) is a major problem in public health in the world.^[1,2]^ COVID-19 has spread throughout China and globally, as a pandemic.^[3,4]^ Up to June, 2020, the number of infections arising to 869,0000 and cause 41, 0000 deaths all over the world.^[5]^ Clinical symptoms of COVID-19 mainly including fever, cough, and fatigue.^[6]^ Disease severity of COVID-19 could be divided into: mild, moderate, severe, critical, and death.^[7]^ Hinder the severity from mild or moderate to severe is top priority for clinicians.^[8]^ In a single-center case series, 26% of patients required admission to the intensive care unit and 4.3% died.^[9]^ Although some previous studies have demonstrated that SARS-CoV-2 infection affects adults and children differently, the data of a systematic meta-analysis on characteristics of children with COVID-19 is still lacking.

In this study, we systematically reviewed relevant published articles about clinical symptoms of COVID-19 pneumonia in children and used meta-analysis methods to analyze the clinical symptoms of COVID-19 pneumonia in children.

## 2 Methods and analysis

### 2.1 Study registration

We tried to plan, perform and report this meta-analysis in comply with Preferred Reporting Items for Systematic Review and Meta-analyses (PRISMA) guideline, and registered in the Registry of Systematic Review/Meta-Analysis (https://www.researchregistry.com/browse-the-registry#registryofsystematicreviewsmeta-analyses/, No. reviewregistry1046). And this study protocol was funded through a protocol registry. This study receives ethics approval from The First Affiliated Hospital of University of South China.

### 2.2 Inclusion and exclusion criteria

In this study, both randomized controlled studies and cohort studies were included. The diagnosis of COVID-19 was confirmed as positive result for nasopharyngeal swab and respiratory pathogen nucleic acid test with high-throughput sequencing or real-time reverse transcriptase polymerase chain reaction (RT-PCR). Diagnostic criteria for COVID-19 severity are based on the CDCP (China) Diagnosis and Treatment of COVID-19. All of the studies about the clinical symptoms of COVID-19 pneumonia in children were included. Exclusion criteria were as follows:

(1) without insufficient data to pool;
(2) case reports;
(3) without gold standard for diagnosis of COVID-19.

### 2.3 Study search

Electronic databases including PubMed, EMBASE, Web of Science, China National Knowledge Infrastructure (CNKI) database, Wanfang Database, and Chinese Biomedical Literature Database (CBM) were searched by 2 reviewers from its inception to June 21, 2020. Search terms included (Mesh “COVID-19” and key words “Novel coronavirus,” “Novel coronavirus 2019,” “2019 nCoV” “COVID-19” “Wuhan coronavirus”), and (Mesh “COVID-19” and keywords “SARS-CoV-2” “Wuhan pneumonia”). A manual search of the references of all the retrieved publications was conducted to identify additional studies. The uniformity between the 2 reviewers was tested using the kappa consistency: fair, 0.40 to 0.59; good, 0.60 to 0.74; and excellent, 0.75 or more.^[10]^

### 2.4 Study selection

**Figure 1:**
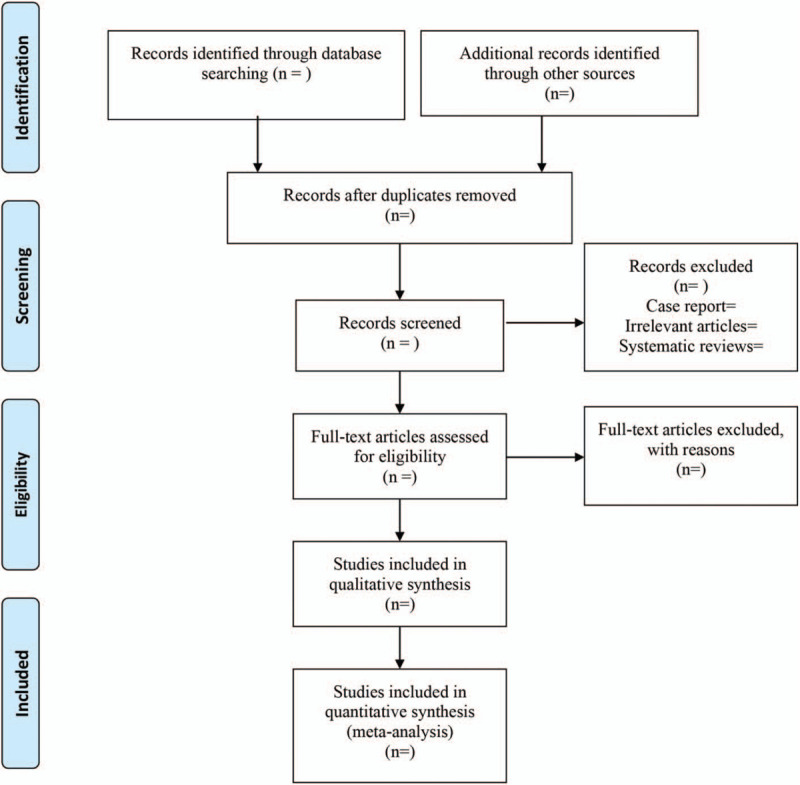
The flow diagram of procedure to select studies.

EndNote X9 (Thomson Reuters, Toronto, Ontario, Canada) was used for literature managing and records literature selection. Study selection was conducted independently by 2 reviewers (Zhengwu Tang and Muzhe Li) and discrepant results were resolved by discussion until a unanimous decision was reached. The study flow chart is presented in Figure 1.

### 2.5 Data extraction

The following information was extracted: the first author, year of publication, number of the patients, mean age of patients, onset time, contact history, and clinical symptoms (fever, cough, sore throat, tachycardia, rhinorrhea, nasal congestion, tachypnea, diarrhea, vomiting, myalgia or fatigue, hypoxemia, and chest pain), clinical laboratory outcomes (white blood cells, C-reactive protein, liver function and renal function). Clinical symptoms in COVID-19 pneumonia in children were collected and recorded in Microsoft Excel (Microsoft Corp., Redmond, WA).

### 2.6 Risk of bias assessment

Two researchers (Wei Chen and Xun Ran) independently assessed the quality of the included trials based on Newcastle-Ottawa quality assessment scale assessment tool.^[11]^ This tool mainly including 3 items: selection, comparability, and exposure. A “
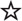
” rating system was used, and scores were ranged from 0 to 9. Studies with a score ≥7 were considered to be of high quality.

### 2.7 Data analysis

Stata 12.0 software (Stata Corp LLC, College Station, TX) was used for meta-analysis. For discontinuous variables, odds ratio was used to assess the effect of severe vs nonsevere COVID-19. All results were presented as forest plot. Heterogeneity was quantified using *I*^2^, with *I*^2^ values >50% representing moderate heterogeneity. To explore sources of heterogeneity, subgroup analysis was performed by age of patients (<60 vs ≥60). Publication bias was ruled out by funnel plot and statistically assessed by Begg test (*P* > .05 as no publication bias).

## 3 Discussion

The aim of this study was to summarize the clinical symptoms of COVID-19 pneumonia in children to provide guidance on disease development. This study has some highlights. First, this is the first systematic review and meta-analysis about the clinical symptoms of COVID-19 pneumonia in children. In addition, we systematically searched the both English and Chinese databases to comprehensively selected the published papers. These methods demonstrate the reliability of our study. Consistency between reviewers was identified by kappa value. Finally, identify the clinical symptoms of COVID-19 pneumonia in children was critically important for clinician to predict accurately of the disease development.

## Acknowledgment

The authors thank the Registry of Systematic Review/Meta-Analysis platform for registry for this meta-analysis.

## Author contributions

**Conceptualization:** Muzhe Li.

**Data curation:** Zhengwu Tang.

**Formal analysis:** Zhengwu Tang, Huiyun Li.

**Validation:** Xun Ran, Zhiwen Chen.

**Visualization:** Wei Chen, Xun Ran, Huiyun Li.

**Writing – original draft:** Muzhe Li, Wei Chen, Xun Ran, Zhiwen Chen.
